# Elucidation of the Mechanism of Action for Metal Based Anticancer Drugs by Mass Spectrometry-Based Quantitative Proteomics

**DOI:** 10.3390/molecules24030581

**Published:** 2019-02-06

**Authors:** Shuailong Jia, Runjing Wang, Kui Wu, Hongliang Jiang, Zhifeng Du

**Affiliations:** 1 Tongji School of Pharmacy, Huazhong University of Science and Technology, Wuhan 430030, China; jsl1993521@163.com (S.J.); wangrjcn@foxmail.com (R.W.); jianghongliang@hust.edu.cn (H.J.); 2 School of Chemistry and Chemical Engineering, Wuhan University of Science and Technology, Wuhan 430081, China; wukui@wust.edu.cn

**Keywords:** anticancer drugs, metal complexes, mechanism, mass spectrometry, quantitative proteomics

## Abstract

The discovery of the anticancer activity of cisplatin and its clinical application has opened a new field for studying metal-coordinated anticancer drugs. Metal-based anticancer drugs, such as cisplatin, can be transported to cells after entering into the human body and form metal–DNA or metal–protein adducts. Then, responding proteins will recognize adducts and form stable complexes. The proteins that were binding with metal-based anticancer drugs were relevant to their mechanism of action. Herein, investigation of the recognition between metal-based anticancer drugs and its binding partners will further our understanding about the pharmacology of cytotoxic anticancer drugs and help optimize the structure of anticancer drugs. The “soft” ionization mass spectrometric methods have many advantages such as high sensitivity and low sample consumption, which are suitable for the analyses of complex biological samples. Thus, MS has become a powerful tool for the identification of proteins binding or responding to metal-based anticancer drugs. In this review, we focused on the mass spectrometry-based quantitative strategy for the identification of proteins specifically responding or binding to metal-based anticancer drugs, ultimately elucidating their mechanism of action.

## 1. Introduction to Metal-Based Anticancer Drugs

**Figure 1:**
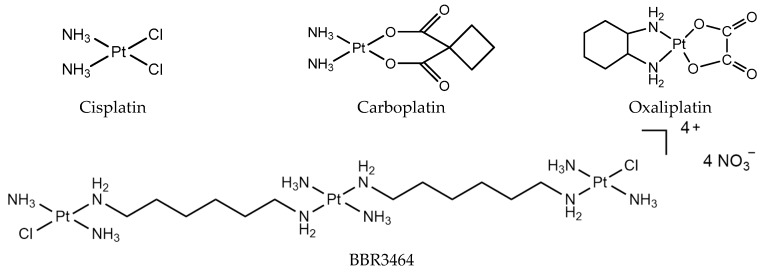
Platinum chemotherapeutic drugs: cisplatin, carboplatin, oxaliplatin, and BBR3464.

As early as the 1960s, Rosenerg first discovered that platinum complexes have an inhibitory effect on tumor cell growth, and used platinum complexes to treat tumors [1]. In the past 30 years, cisplatin, carboplatin, nedaplatin, oxaliplatin, and lobaplatin (Figure 1) have been successfully developed and used for the clinical treatment of cancer. In particular, cisplatin, oxaliplatin, and carboplatin are used worldwide as anticancer drugs. To date, all of the clinically used platinum drugs contain a single Pt^II^ center with two exchangeable ligands in cis geometry. The interaction of these drugs with cellular biomolecules such as sulfur-containing glutathione and metallothionein can deactivate them before reaching their pharmacological target, DNA [2,3]. After platinum drugs enter into the body, the intracellular Cl^−^ concentration is low, and the drug is easily hydrolyzed to form an active molecule. The active hydrolysate has a positive charge, and is electrostatically attracted by DNA, which is a negatively charged genetic material located in the nucleus [4]. Binding to DNA forms DNA intra-strand cross-linking, inter-strand cross-linking, and DNA protein cross-linking. The formation of DNA cross-linking affects DNA strand synthesis, replication, and ultimately leads to cell death [5,6,7,8,9].

The study of the mechanism of classical platinum drugs has helped chemists develop new platinum drugs. Polynuclear platinum complexes (PPCs) are a new class of platinum anticancer complexes, which are structurally different from cisplatin, and exhibit a different mode of DNA binding, such as the phosphate clamp DNA binding mode of substitution-inert PPCs [10]. Especially, conformational changes induced by long-range inter-strand and intra-strand cross-links are distinctly different from those induced by mononuclear platinum complexes [11]. The prototype of this class, BBR3464 [{*trans*-PtCl(NH_3_)_2_}_2_{µ-*trans*-Pt(NH_3_)_2_(H_2_N(CH_2_)_6_NH_2_)}]^4+^ (Figure 1), is the only platinum compound without two exchangeable ligands in cis, and has reached Phase II clinical trials [12]. It is cytotoxic in cisplatin-resistant cell lines, and shows high efficacy in p53 mutant tumor cells [13]. BBR3464 can be deactivated in human plasma [14,15].

In addition to PPCs, there are also other *trans*-platinum complexes. Early studies have suggested that transplatin is inactive, but recent studies have found that some *trans*-platinum complexes have good in vitro and in vivo anti-tumor activity. The anti-tumor mechanism of this complex is still unclear. Generally, although DNA has long been believed to be the major target of platinum anticancer drugs, several proteins/enzymes have recently been proposed to be involved in the action of platinum complexes [16].

**Figure 2:**
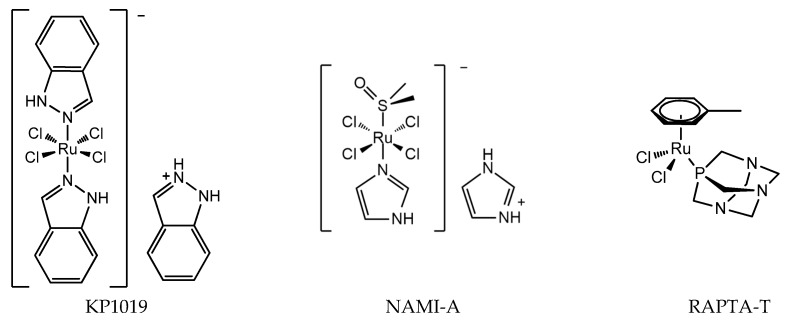
Chemical structure of ruthenium anticancer drugs: Ru^III^ compounds indazolium *trans*-[tetrachloridobis(1*H*-indazole)ruthenate(III)] (KP1019), imidazolium *trans*-[tetrachlorido(DMSO)(1*H*-imidazole)ruthenate(III)] (NAMI-A), RAPTA-T.

The discovery of the anticancer activity of cisplatin and its clinical application has triggered the study of metal-coordinated anticancer drugs; however, the serious side effects and intrinsic or acquired drug resistance of cisplatin largely limited its further clinical application. This has led medicinal chemists to explore other metal-based anticancer candidates, for example Ti, Os, and Ir complexes, to circumvent the problems associated with cisplatin administration [17,18,19,20]. A number of non-platinum complexes have entered clinical trials. Ruthenium compounds are regarded as promising alternatives to anticancer platinum drugs based on several advantages, for example, ruthenium compounds have lower toxicity and less drug resistance [21,22]. The most important developments comprise the clinically tested Ru^III^ compounds indazolium *trans*-[tetrachloridobis(1*H*-indazole)ruthenate(III)] (KP1019), imidazolium *trans*-[tetrachlorido(DMSO)(1*H*-imidazole)ruthenate(III)] (NAMI-A), and Ru^II^ compounds RAPTA (Figure 2). The structure of KP1019 was slightly modified due to solubility reasons, and was renamed as NKP-1339 (also IT-139). NKP-1339 is currently in clinical trials, and obtained orphan status from the Food and Drug Administration (FDA) in 2017. While the clinical trial for NAMI-A has been abandoned. Ru^III^ has a wide range of coordination numbers and geometries, as well as accessible redox states, which offer the medicinal chemists a wide spectrum of reactivities that can be exploited. RAPTA complexes are a promising class of organometallic Ru^II^ compounds that inhibit processes related to metastasis in vitro and exhibit pronounced antimetastatic activity in vivo, but only low antiproliferative activity [23,24]. Moreover, it has been shown that RAPTA compounds preferentially bind to proteins, even in the presence of DNA [25,26].

**Figure 3:**
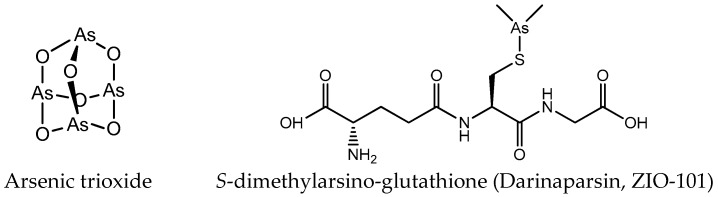
Chemical structure of arsenic anticancer drugs.

Except platinum and ruthenium, arsenic and gold complexes are also used as anticancer agents. As^III^ and As^V^ are the main oxidation states of arsenic. The cytotoxic activity of arsenic compounds in the trivalent state is strongly associated with the enhanced production of reactive oxygen species (ROS) [27,28]. The biological activity of pentavalent arsenic is mainly based on substitution for phosphate (e.g., in ATP) [29]. Amongst the various forms of arsenicals, the greatest clinical success has been the one of arsenic trioxide (ATO; As_2_O_3_, Trisenox^®^) (Figure 3) in the treatment of hematological cancers, especially acute promyelocytic leukemia (APL) [30,31]. Arsenic trioxide forms inorganic As(OH)_3_ in an aqueous environment, and this form can be transported intracellularly via aquaglyceroporin channels due to its similarity to glycerol [32]. In addition to arsenic trioxide, other organic and inorganic arsenic agents are undergoing clinical trials for hematological malignancies, such as *S*-dimethylarsino-glutathione (Figure 3) [33].

**Figure 4:**
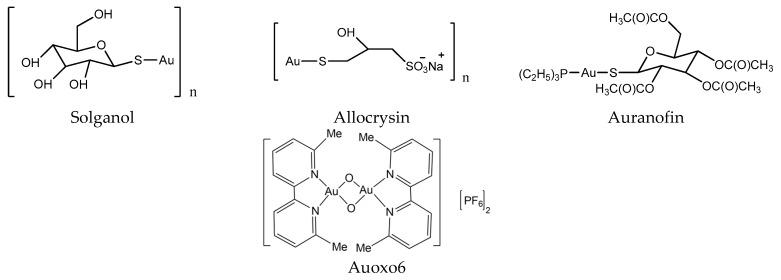
Chemical structure of gold anticancer drugs.

In the early days, gold compounds were mainly used for the treatment of rheumatoid arthritis (RA). Auranofin (Ridaura) was approved by the FDA for the treatment of RA in 1985 (Figure 4) [34]. Au compounds can be considered as prodrugs, and require activation (achieved by ligand exchange reactions) before they can develop their full pharmacological potential [29]. Several Au compounds are found to resolve the resistance of platinum compounds confirming different mechanisms of action [35]. It seems that the main targets of Au compounds are proteins rather than DNA, since scientists found the selenoprotein thioredoxin reductase plays a crucial role in the biological actions of gold compounds and acts as a major and general receptor for gold compounds, which can interact with specific thiol-containing and seleno-containing peptide moieties [36,37].

In addition to the above-mentioned metal drugs, many other metal complexes can be potentially used for the treatment of cancer, such as vanadium (V), rhodium (Rh), zinc (Zn), and cobalt (Co) [26]. Generally, metal-based anticancer drugs have high affinity for sulfur-containing biomolecules, such as proteins with Cys and Met residue. Thus, protein may play an important role in the mechanisms of those drugs such as drug resistance, toxicity, and metabolism. A comprehensive investigation of the interaction between metal-based anticancer drugs and their binding proteins or cell will further our understanding about the pharmacology of cytotoxic anticancer drugs from a molecular level. Mass spectrometry-based quantitative strategy has been widely used for the identification of proteins specifically responding or binding to metal-based anticancer drugs.

## 2. Quantitative Proteomics: General Remarks

Proteomics is the large-scale study of proteins, with a particular emphasis on their structures and functions. Proteins form a crucial part of the living organisms, as they are the main components of the metabolic and signaling pathways of cells while playing very important structural roles. Mass spectrometry is widely used in proteomic research because of its various advantages, including high sensitivity, high throughput, and good compatibility. It is even more powerful when combined with other separation techniques such as capillary electrophoresis (CE) and liquid chromatography (LC). Among the mass spectrometric ion source, ESI and MALDI are the most commonly used. Briefly, ESI generates multiply charged ions for biomolecules, while MALDI yields mostly singly charged pseudomolecular ions of analytes. ESI-MS can be easily hyphenated with separation techniques, whereas MALDI-TOF-MS cannot be directly combined to chromatographic methods; thus, MALDI-TOF-MS is usually accompanied with two-dimensional (2D) gel electrophoresis in proteomics, while ESI-MS is often coupled with nano-LC in proteomics to achieve higher sensitivity. Consequently, both ion sources are utilized for proteomic research.

Most of the mass spectrometers that are available offer one or more fragmentation methods that are used to provide information about the structure and composition of the ion of interest. Collision-induced dissociation (CID) is the most frequently employed fragmentation technique in current MS-based proteomics. In CID, selected peptides are subjected to collisions with inert gas molecules such as helium and nitrogen [38]. HCD is another fragmentation method, which is available for the LTQ Orbitrap [39]. In HCD, ions are fragmented in a collision cell rather than an ion trap, and then transferred back through the C-trap for analysis in the Orbitrap [40]. Besides CID and HCD, electron-based approaches such as electron capture dissociation (ECD) and electron transfer dissociation (ETD) are also used in MS-based proteomics as fragmentation techniques. The mechanism of electron-driven fragmentation techniques is fundamentally different from CID [41].

There are two general strategies applied in proteomic, which are called “bottom–up” and “top–down”. Top–down describes the fragmentation of intact proteins without enzyme digestion. This approach is limited to small and pure proteins, while bottom–up involves enzymatic digestion of the analytes prior to fragmentation, and requires more sample preparation before data acquisition. A variety of chromatographic separation and enrichment methods could be used to achieve better peptide coverage and more identification. Owing to the introduction of enzymatic digestion, this strategy is well suited for investigating lager proteins and protein mixtures. For the quantitative proteomics research of biological systems, the “bottom–up” approach is more widely used.

**Figure 5:**
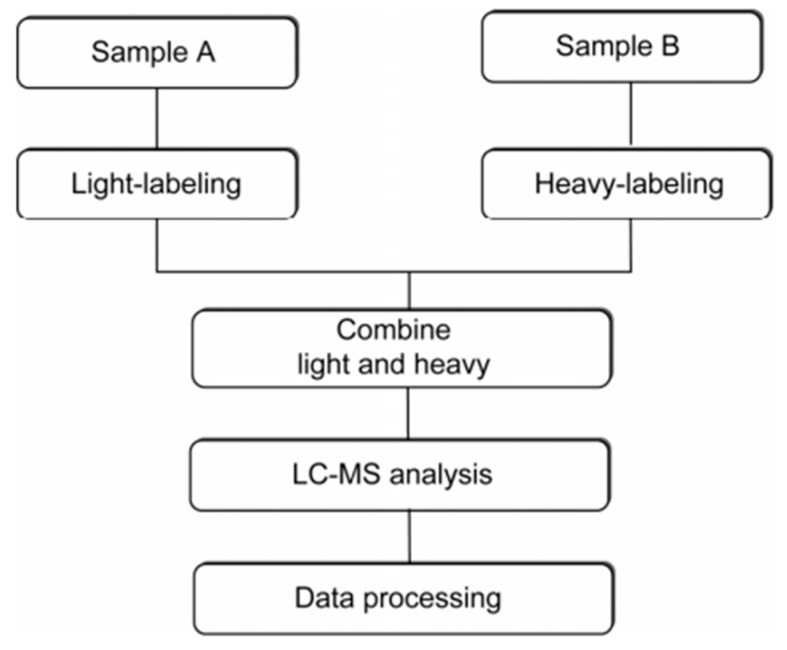
The workflow of stable isotope labeling-based quantitative proteomics. This figure is adapted from reference [44].

Current methods for protein quantitation can be classified into two main categories: stable isotope labeling and label-free quantitation. Stable isotope labeling can introduce isotopically labeled forms into target components for relative quantification. The light labeled and heavy labeled proteins are mixed proportionally before LC/MS analysis, and the peak area ratio of ion pairs generated by isotopically labeled analytes is used to quantify the components in the sample [42]. The workflow is shown in Figure 5. ICAT, TMT, iTRAQ, and SILAC belong to this category, and they are commonly used in quantitative proteomics research. A comparison of those methods is shown in Table 1.

**Figure 6:**
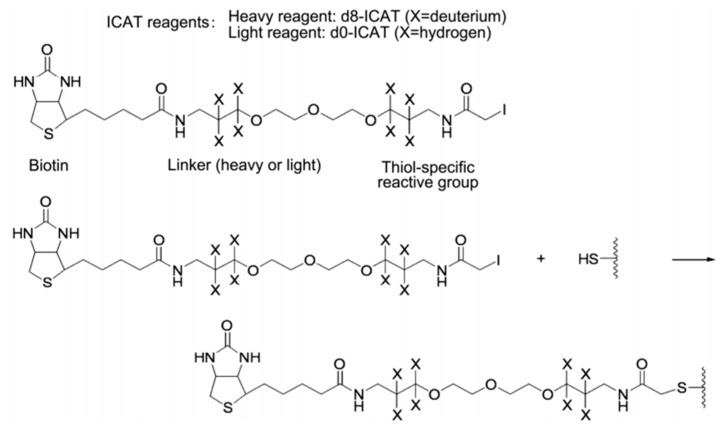
Structure and reaction mechanism of the ICAT reagent. This figure is adapted from reference [57].

The ICAT reagent mainly consists of three parts: the first part is the affinity tag composed of biotin, which is used to separate ICAT-labeled peptide. The second part is the linker, and it is used to introduce the stable isotope. The third part is the reactive group that specifically binds to the thiol group of a cysteine residue in a peptide. The ICAT reagent exists in two forms—light (contains no deuterium) and heavy (contains eight deuterium atoms) form—and the mass difference among them is exactly eight Da (Figure 6). After the isotopic label is introduced into the peptide, the response intensity of two labeled forms for the same peptide is compared by MS. ICAT can analyze most proteins in cells, tissues, body fluids, etc., with good compatibility, but it should be noted that this method is only applicable to proteins containing cysteine [43,54,55,56].

**Figure 7:**
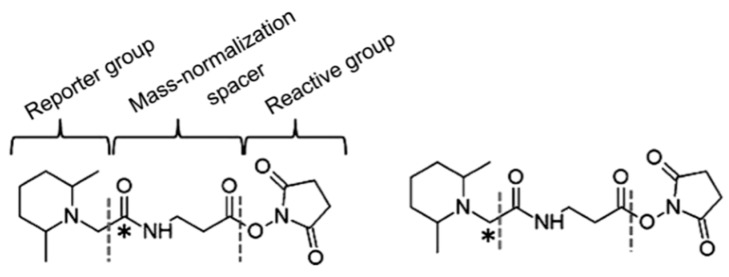
The chemical structure of two-plex tandem mass tag (TMT) reagents. This figure is adapted from reference [58].

In order to solve the shortcomings of the ICAT reagent, Thompson et al. synthesized TMT (tandem mass tags) reagent. The chemical structure of the commercial TMT agent is shown in Figure 7. It consists of a mass reporter region, a cleavable linker region, a mass normalization region, and a reactive group. The reactive group can specifically bind to the −NH_2_ group of the peptide. When utilized for the relative quantification of multiple sets of protein samples, it enables the isotopically-labeled forms of target molecules to have identical chromatographic behavior and primary MS characteristic. The different label forms of target peptides are dissociated in the cleavable linker region and form different reporter ions for mass spectrometry (MS/MS) detection. Therefore, the relative content of target protein in different samples can be determined after comparing the intensity of reported ions.

**Figure 8:**
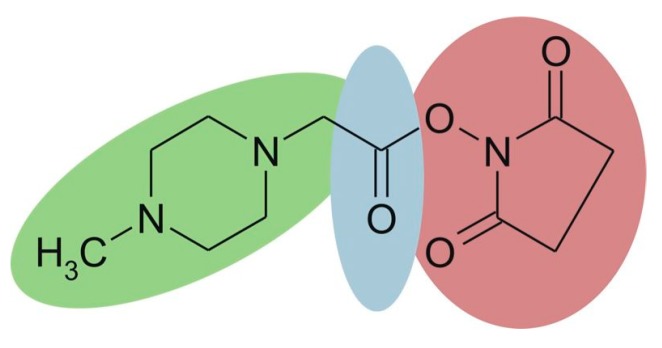
Chemical structure of the iTRAQ™ reagent. This figure is adapted from reference [61].

iTRAQ is based on the same labeling strategy as TMT. The reagent consists of three parts (Figure 8), and the four available tags have identical overall mass. For four-plex iTRAQ reagents, the reporter group (green, N-methylpiperazine) mass is 114, 115, 116, and 117, respectively. The balance group (blue, carbonyl group) has a mass of 31 Da, 30 Da, 29 Da, and 28 Da accordingly. The reactive group (red, NHS ester) selectively reacts with the amino group of the peptide (the *N*-terminus and amino groups of the side chain). After LC-MS analysis, proteins are quantified by the relative intensity of the reporter group in MS/MS spectra. The iTRAQ technique has been widely used for the quantitative study of proteins due to its high accuracy and precision. What’s more, it is able to simultaneously and quantitatively analyze up to eight samples [59,60]. However, iTRAQ reagents are expensive, and can significantly increase the cost of research.

**Figure 9:**
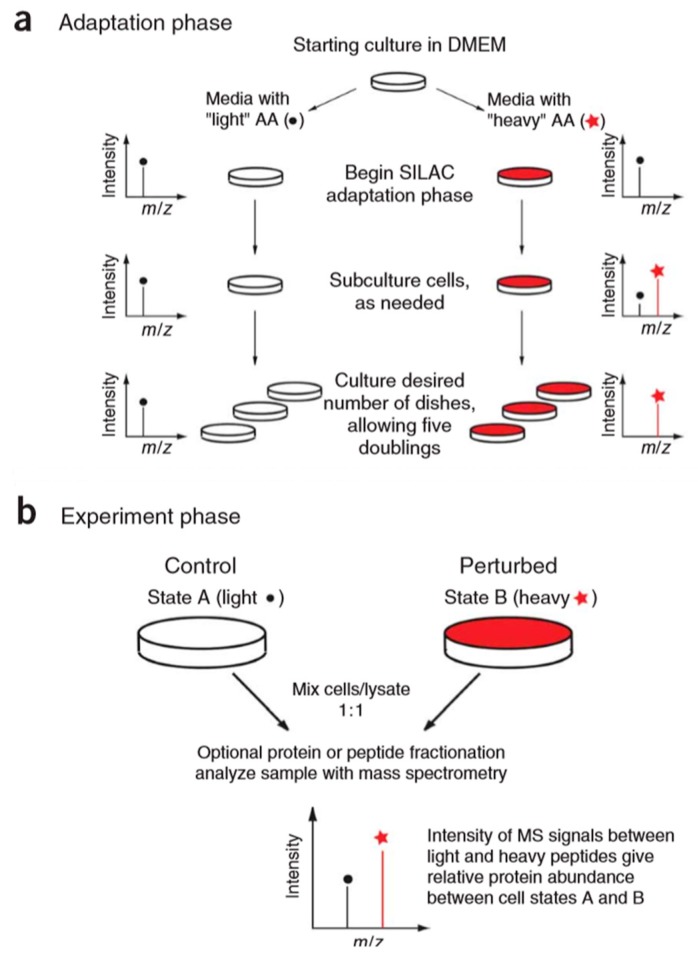
Overview of stable isotope labeling by amino acids in cell culture (SILAC) protocol. The SILAC experiment consists of two distinct phases: an adaptation (**a**) and an experimental (**b**) phase. This figure is adapted from reference [64].

The stable isotope labeling methods mentioned above are all in vitro methods, and the typical example of the in vivo method for the relative quantitation of proteins is the stable isotope labeling by amino acids in cell culture (SILAC) [62]. The basic principle of this technique (Figure 9) is to add light and heavy isotopically-labeled essential amino acids (usually lysine and arginine) to the cell culture medium. After five to six doubling cycles, the amino acids of the newly synthesized protein in the cell are almost completely labeled with stable isotopes. Therefore, the accurate quantification of proteins can be achieved based on the peak intensity or area ratio of the two isotopically labeled peptides in the mixed sample. SILAC technology is able to mix samples at the protein level, which can effectively avoid the quantitative error caused by subsequent enzymatic digestion. It has high labeling efficiency and high quantitative accuracy. However, the presence of isotope-labeled arginine metabolism and the proline formation lead to low labeling efficiency and decreased quantification accuracy [63]. At the same time, this technique is only applicable to the cultured cell, and cannot be applied to samples such as tissues and body fluids, which are commonly used in medical research.

Label-free quantitative proteomics (LFQP) is based on spectral counting and peak intensity for comparative analysis of the abundance of proteins. It provides a straightforward option for the large-scale analysis of biological samples. In contrast to label-based methods, LFQP mainly quantifies the number of identified peptides and the area of primary mass spectral peak by calculating the peptide fragment matching [65,66]. LFQP is cost-effective, and does not require expensive isotope reagents. In addition, LFQP is not time-consuming compared with some label-based methods that require tedious labeling steps [67,68]. Due to the aforementioned reasons and the increase of analytical capabilities of LC-MS/MS instrumentation in terms of resolution, accuracy, and sensitivity, LFQP has gained more acceptance in biomedical research. The characteristic peptides of each protein can be used for quantification [69], which can effectively improve the accuracy of non-labeled quantification. However, the label-free approach requires more rigorous analytical measurements and statistical validation compared with isotope-coded measurements.

As we can see, both labeling and label-free approaches have their own advantages and limitations (Table 1). More and more methods are developed for quantitative proteomic analysis in recent years. Those are the foundation for studying the mechanism of metal-based anticancer drugs.

## 3. Application of Quantitative Proteomics for Elucidation of the Mechanism of Metal-Based Anticancer Drugs

### 3.1. Proteomic Analysis of Cellular Responses to Metal-Based Anticancer Drugs

It’s very important to monitor, in real time, the proteomic responses of cells to cytotoxic metallodrugs, as such responses might provide valuable information on the mechanism of action of the drug itself, and highlight which metabolic or signaling pathways of the cell are primarily affected and/or activated. If the damage is too intense to be repaired, specific biochemical pathways will be triggered, ultimately leading to cell apoptosis [70]. A number of studies utilizing this kind of strategy have appeared in recent literatures.

Cho et al. exploited the proteomic method based on a label-free quantitation strategy to study the cisplatin-induced hepatotoxicity [71]. Results showed that 76 proteins were up-regulated, and 19 proteins were down-regulated. The up-regulated proteins in the cisplatin-treated group include FBP1 (fructose 1,6-bisphosphatase 1), FASN (fatty acid synthase), CAT (catalase), PRDX1 (peroxiredoxin-1), HSPD1 (60-kDa heat shock protein), MDH2 (malate dehydrogenase 2), and ARG1 (arginase 1). Down-regulated proteins in the cisplatin-treated group include TPM1 (tropomyosin 1), TPM3 (tropomyosin 3), and CTSB (cathepsin B), which were further confirmed by Western blot analysis. Subsequent pathways analysis revealed that drug metabolism, fatty acid metabolism, glycolysis/TCA cycle, urea cycle, and inflammation metabolism were involved in cisplatin-induced hepatotoxicity.

Cytotoxic gold-based complexes have different modes of action from cisplatin. It was found that auranofin and Auoxo6 are more active than cisplatin against both the cisplatin-sensitive A2780/S and resistant A2780/R cell lines. Guidi et al. reported the use of 2-DE and MS analysis to find the molecular mechanisms through which auranofin and Auoxo6 caused their biological effects [52]. 2-DE combined with MALDI-TOF analyses showed that 10 proteins were down-regulated, and one protein was up-regulated in A2780/R cells treated with auranofin versus controls. For Auoxo6, 12 proteins were down-regulated and four proteins were up-regulated. After investigation of the altered proteins, they proposed that auranofin mostly acts by altering the amount of proteasome proteins, while Auoxo6 mainly modifies proteins related to mRNA splicing, trafficking, and stability. Interestingly, thioredoxin-like protein 1, which is involved in oxidative stress defense, was greatly reduced after treatment with both gold compounds.

**Figure 10:**
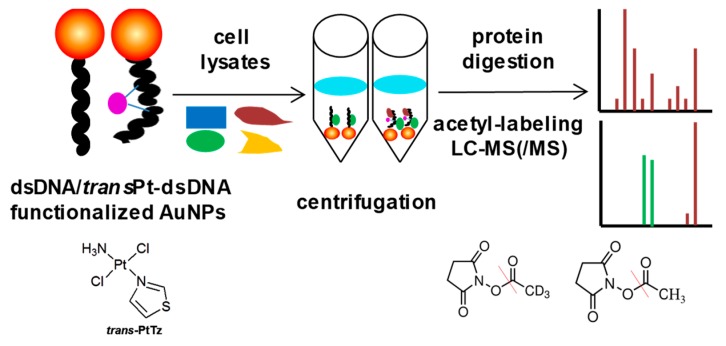
Overview of the MS-based proteomic strategy to study the cellular proteins responding to damaged DNA by platinum drugs. This figure is adapted from reference [72].

The characteristics of high anticancer efficiency of cisplatin and the clinical inactivity of its trans isomer (transplatin) have been considered a paradigm for the classical structure-activity relationships of platinum drugs. However, some new analogues of transplatin exhibit activity in cisplatin-resistant tumor cells, but the mechanism behind this activity is unknown. An MS-based proteomic strategy combined with functionalized gold nanoparticles as affinity probes was developed to study the cellular proteins responding to damaged DNA by cisplatin and *trans*-PtTz (Figure 10) [72]. To exclude nonspecific binding proteins to the platinated DNA, the negative probe with native double-stranded DNA on gold nanoparticles was utilized as a control. As a result, the well-known protein HMGB1 was identified as a binding partner of platinated DNA by cisplatin, and the nuclear protein positive cofactor PC4 was found to bind specifically with cross-linked DNA by *trans*-PtTz, which will shed light on the mechanism of this active transplatinum complex.

Palladium (Pd)-based compounds have gained the interest of many researchers, as they exhibit similar coordination chemistry and better solubility compared with platinum drugs [73]. [Pd(sac)(terpy)](sac)·4H_2_O (sac = saccharinate, and terpy = 2,2′:6′,2″-terpyridine), a palladium-based compound, was reported to be more potential than cisplatin in breast cancer cells. To figure out the mechanism of action, Adiguzel et al. performed nanoLC-MS/MS analysis to investigate the global proteomic changes after treatment of this Pd(II) complex [48]. Eventually, 681 proteins were identified, among which 335 protein groups were quantified through the label-free quantitative method. Furthermore, 30 differentially expressed proteins were identified between drug-treated cells and untreated cells. These proteins were involved in the regulation of apoptosis, proliferation, protein degradation, and DNA repair, etc. Ingenuity pathway analysis revealed that the involved pathway appeared as the protein ubiquitination; therefore, apoptosis is the mechanism of cell death in response to Pd(II) treatment, as a significant increase in ubiquitination was identified. Finally, they proposed that the mode for the cytotoxic action of the Pd(II) complex was that cells initially attempt to repair Pd(II)-induced damage, yet prolonged damage or exposure of higher doses lead to protein degradation and apoptosis in cancer cells.

### 3.2. Analysis of the Molecular Basis of Platinum Resistance through Comparative Proteomic Analysis of Pt Sensitive versus Pt-Resistant Cell Lines

There are lots of studies about the molecular basis of platinum drugs resistance. Indeed, acquired resistance is often the reason for treatment failure, and therefore attracts great interest. Understanding the molecular mechanism of resistance might help to circumvent it. Thus, a conspicuous number of proteomic studies were specifically devoted to this issue.

A quantitative proteomic screening was performed to identify the proteins that are differentially expressed in drug-resistant cell lines through which the mechanisms involved in cisplatin resistance may be found out [46]. The SILAC approach with nano-LC-MS was employed in this experiment. As a result, a total of 856 proteins, among which 374 proteins were differentially expressed between the cisplatin-resistant cell (HeLa/CDDP) and sensitive cells (HeLa) were identified. The expression of a few key proteins, including CD44, DDB-1, DJ-1, and XRCC5, were confirmed by Western blotting, which was highly consistent with the proteomic analysis. A further protein interaction network based on the differentially expressed proteins was constructed, and finally, the biological pathways, including carbohydrates, energy-producing, regulation of apoptosis, and protein folding were involved in the drug resistance.

A number of studies have found that mitochondria was correlated with the cisplatin resistance. Mitochondrial DNA and membrane proteins were reported as preferential targets of cisplatin. It was also shown by several groups that mitochondria impairment appeared to play an important role in the platinum resistance of ovarian cancer cells. Using 2D DIGE integrated with MALDI-TOF-MS, Dai et al. investigated the mitochondrial proteins difference between platinum-sensitive human ovarian cancer cell lines (SKOV3 and A2780) with that of four platinum-resistant sublines (SKOV3/CDDP, SKOV3/CBP, A2780/CDDP, and A2780/CBP) [51]. Through a 2D DIGE experiment, 236 spots were identified, among which 128 spots were down-regulated in platinum-resistant cells, and 108 spots were up-regulated in these resistant cells. Eleven spots that had more than threefold changes in platinum-resistant cells compared with platinum-sensitive cell lines were analyzed and verified by MALDI-TOF-MS. Five of the proteins, ETF, PRDX3, PHB, ATP-a, and ALDH, were identified. ATP-a, PHB, and PRDX3 have been validated as mitochondrial proteins of ovarian cancer cells, and they were further confirmed through immunoblotting. The expressions of ATP-a, PHB, and PRDX3 were further validated in the clinical ovarian cancer sections; as a result, a significant difference existed in PHB expression between the sensitive group and the resistant group, demonstrating that PHB might be a correlative candidate protein for platinum resistance in the mitochondria of ovarian cancer cells.

Using the ICAT approach, Stewart et al. profiled the nuclear, cytosolic, and microsomal fractions of IGOV-1 (cisplatin-sensitive) and IGOV-1/CP (cisplatin-resistant) ovarian cancer cell lines [43]. A total of 1117 proteins were identified and quantified, among which 121 proteins were expressed differentially in cisplatin-resistant cell lines compared with the sensitive ones. Sixty-three proteins were overexpressed in cisplatin-sensitive cells, and 58 proteins had low expression in these cells. Among the 63 overexpressed proteins, several proteins were overexpressed at least fivefold in resistant cells, including cell recognition molecule CASPR3 (13.3-fold), S100 protein family members (8.7-fold), junction adhesion molecule Claudin 4 (7.2-fold), and CDC42-binding protein kinase (5.4-fold). Other proteins exhibited low expression for at least fivefold in resistant cells, including hepatocyte growth factor inhibitor 1B (13.3-fold) and programmed cell death 6-interacting protein (12.7-fold). They also compared the expression of mRNA with that of protein in a subset of 92 highly differentially expressed proteins, and the expression level of 37 proteins are in the same direction with that of mRNA, and 55 are discordant, possibly reflecting the post-transcriptional control of protein expression. By Gene Ontology (GO) analysis, many processes, including RNA splicing, processing, and DNA replication were increased in cisplatin-resistant cells. The increased activities of these biological processes may lead to faster repairs of cisplatin-induced DNA damages, thus resulting in a resistant phenotype. They also found that three pathways (glycolysis, the interleukin signaling pathway, and the PI 3-kinase pathway) were significantly up-regulated in cisplatin-sensitive cells, which were involved in cell apoptosis.

Cisplatin-based chemotherapy is currently used for bladder cancer (BC), but it lacks efficiency on patients who have acquired or developed resistance. In order to reveal the molecular mechanisms underlying this resistance, Jung et al. carried out a multidimensional proteomic analysis on cisplatin-sensitive (T24S) and resistant (T24R) T24 human BC cell lines [45]. It was reported that the aberrant expression or mutations of the EGFR family are related to the carcinogenesis of bladder cancer (BC); therefore, the temporal changes in protein abundance and phosphorylation in T24S and T24R cells after EGF stimulation were also investigated. Sixplex TMT reagents were used to label peptide samples. Consequently, the global proteome profiles in both T24R and T24S cells changed slightly. Whereas, phosphoproteome in T24S cells changed more than T24R cells, which revealed that T24S cells were impacted more greatly than T24R cells by EGF stimulation. The analysis of altered proteins revealed associations of cisplatin resistance with DNA damage, repair, and cell cycle regulation, which is consistent with previous reports [74]. Several key regulators linked to cisplatin resistance were confirmed. These results are promising, but need more clinical specimens for phosphorproteomic analysis.

Neuroblastoma is a challenging childhood malignancy with a very high percentage of patients relapsing after the acquisition of drug resistance. In order to investigate the molecular pathways involved in the drug resistance of neuroblastoma, Piskareva et al. characterized three cisplatin-sensitive/resistant cell line pairs using the label-free quantitative method [47]. As a result, 46/72, 68/43, and 34/63 proteins were found to be up-regulated and down-regulated for the three cell line pairs, respectively. Differentially-expressed proteins for each individual cell line pair were used to analyze the molecular and cellular functions that were involved through ingenuity pathway analysis. Known mutual interactions among differentially expressed proteins for each cell line pair were used to construct protein networks. Consequently, four proteins were in common across these networks, including betatubulin (TUBB), beta-actin (ACTB), vimentin (VIM), and 78 kDa glucose-regulated protein (HSPA5). Pathways analysis suggested that the epithelial-to-mesenchymal transition is a feature during the development of drug resistance in neuroblastoma.

### 3.3. Mass Spectrometry-Based Quantitative Proteomics for Identification of Target Proteins for Metal-Based Anticancer Drugs

Target identification utilizing photoaffinity groups together with clickable moieties recently emerged as a useful strategy to identify macromolecular binding partners for small organic molecules, but it was seldom used for the target identification of anticancer metal complexes. Fung et al. developed a chemical probe for the target identification of [Au^III^(C^N^C)(NHC)]OTf by introducing a small photoaffinity diazirine group and a clickable alkyne moiety on NHC [53]. HeLa cells were treated with the probe, and then irradiated followed by a click reaction; eventually, six photoaffinity-labeled proteins were identified by gel electrophoresis accompanied by MALDI-TOF-MS, and these proteins were also found in the protein samples from NCI-H460 and HCT116 cells treated with the same probe. The six proteins were identified as mitochondrial heat shock protein 60 (HSP60), vimentin (VIM), nucleoside diphosphate kinase A (NDKA), nucleophosmin (NPM), nuclease-sensitive element binding protein (Y box binding protein, YB-1), and peroxiredoxin1 (PRDX1) by MALDI-TOF-MS/MS analysis. Except for YB-1, other proteins were verified by HPLC-LTQ-Orbitrap MS analysis with high confidence. Besides, gold(III) meso-tetraphenylporphyrin is notable for its high stability in biological environments and potent in vitro and in vivo anticancer activities. Extensive chemical biology approaches, including photoaffinity labeling, click chemistry, chemical proteomics, and the SILAC technique were used to find the protein target of gold(III) porphyrins [35]. Compelling evidence revealed that heat-shock protein 60 (Hsp60), a mitochondrial chaperone and potential anticancer target, is a direct target of gold(III) porphyrins in vitro and in cells. Structure-activity studies with a series of non-porphyrin gold(III) complexes and other metalloporphyrins revealed that Hsp60 inhibition is specifically dependent on both the gold(III) ion and the porphyrin ligand.

**Figure 11:**
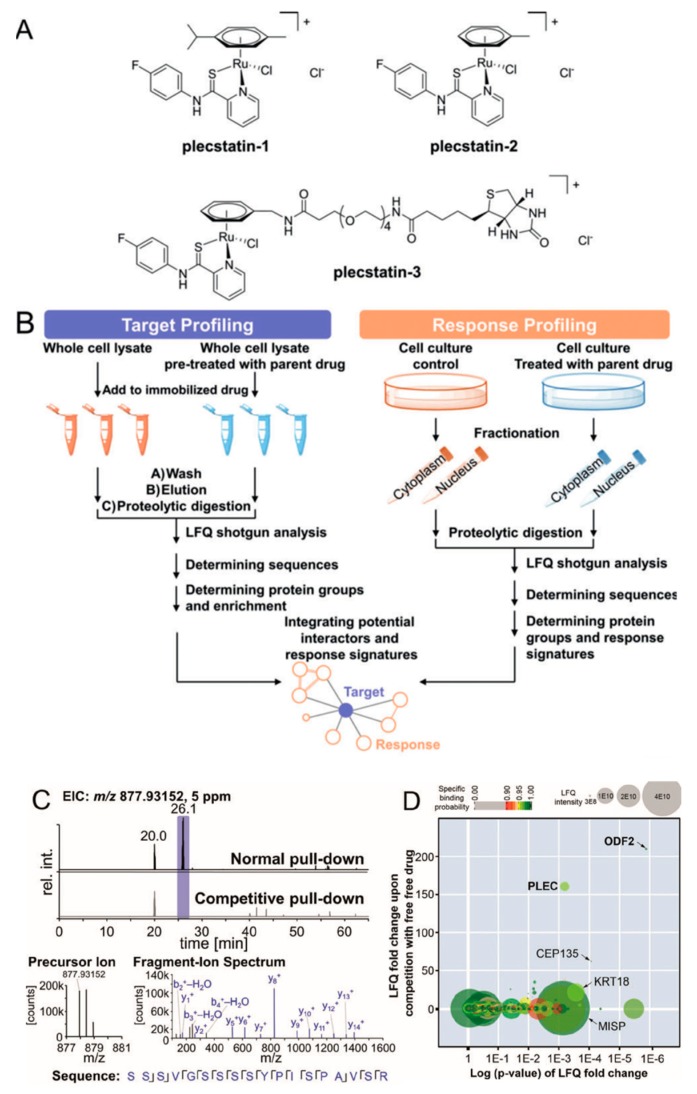
Potential protein targets of ruthenium(arene) pyridinecarbothioamides (plecstatins) were obtained by integrated target-response profiling. (**A**) Chemical structures. (**B**) Schematic representation of the integrated target-response profiling workflow. (**C**) Selected chromatograms and mass spectra of a plectin-specific tryptic peptide. (**D**) Plot of the target profiling experiment. PLEC: plectin; ODF2: outer dense fiber protein 2. This figure is adapted from reference [49].

Organometallic anticancer agents often require ligand exchange for their anticancer activity, which is generally believed to possess low selectivity for potential cellular targets. However, Meier et al. found an unexpected target selectivity of a ruthenium(arene) pyridinecarbothioamide (plecstatin) (Figure 11). They utilized a label-free quantitative method to seek out the potential target of plecstatin. To address nonspecific interactions, a competition experiment was conducted by pretreatment with drug [49]. As a result, roughly 400 proteins were identified, among which only outer dense fiber protein 2 (ODF2, 210-fold) and plectin (PLEC, 160-fold) were considered as potential targets with high enrichment factors. The latter one is a scaffold protein and cytolinker with pronounced effects on the organization of non-mitotic microtubules, which was considered as an unexpected target for plecstatin. Moreover, non-mitotic microtubules are an underappreciated drug target, and their disturbance by plectin-targeting agents affects the motility of cancer cells, which may develop a promising anticancer strategy.

RAPTA compounds have attracted researchers’ attention, as their modes of action are substantially different from commonly used platinum-based chemotherapeutics. In this case, drug pull-down combined with affinity chromatography, mass spectrometry-based proteomics, and bioinformatics were utilized to figure out the target proteins of antimetastatic agent RAPTA in ovarian cancer lysate [50]. A competitive experiment using an acetylated RAPTA analogue was carried out to remove the non-specific binding proteins. Pull-down experiments without competitive experiments resulted in the identification of a total of 184 proteins. After comparing data with the competitive experiment, the number of high-affinity proteins decreased to 29, which can be classified into four types: extracellular proteins, cell cycle-regulating proteins, histone-related proteins, and ribosomal proteins. Among the 29 proteins, 15 proteins were found to be cancer-related. What’s more, the identified proteins, including the cytokines midkine, pleiotrophin, fibroblast growth factor-binding protein 3, guanine nucleotide-binding protein-like 3, and FAM32A were consistent with the hypothesis that the antiproliferative activity of RAPTA compounds is due to the induction of a G2/M arrest and histone proteins [75].

## 4. Conclusion Remarks

In recent years, metal-based anticancer drugs have played an important role in the clinical chemotherapy of cancer, especially platinum-based anticancer drugs represented by cisplatin. The adverse effects and acquired resistance of platinum-based anticancer drugs triggered researchers to exploit novel anticancer metallodrugs and figure out the underlying mechanism of anticancer activity and drug resistance.

Initial studies identified DNA as the primary target of platinum-based drugs, but current research has revealed that metal-based drugs can also bind with proteins, which was correlated with their activity, drug resistance, toxicity, and metabolism. Proteomics is the large-scale study of proteins, and mass spectrometry has been widely used in proteomic research because of its high sensitivity, high throughput, and good compatibility. Current methods for protein quantitation include stable isotope labeling and label-free quantitation, which have been widely used for the studies of target proteins and cellular response proteins for metal-based anticancer drugs. The altered expression of proteins found by quantitative proteomics revealed that several metabolic and signaling pathways, for example protein ubiquitination, were involved in the mechanism of metal drugs. Cellular protein targets identified through quantitative proteomics pinpointed the molecular pharmacology of metal-based anticancer drugs, such as Hsp60 for gold (III) porphyrins. Therefore, mass spectrometry-based quantitative proteomics becomes a powerful tool to elucidate the mechanism of action for metal-based anticancer drugs, which will facilitate the design of more efficient anticancer drugs.

## Figures and Tables

**Table 1:** Comparison of commonly used methods in quantitative proteomics.

Quantitation Methods	Advantages	Disadvantages	Metal Drugs Investigated by Each Method
ICAT	Procedure is easy.	Only two samples can be labeled, which is only applicable to proteins containing cysteine.	Cisplatin [43,44]
TMT	Quantification on multiple sets of protein samples.	Expensive	Cisplatin [45]
iTRAQ	Quantification on multiple sets of protein samples.	Expensive	Cisplatin [44]
SILAC	Applicable to cultured cell.	It cannot be applied to samples such as tissues and body fluids. Expensive, time-consuming, and complicated.	Cisplatin [46] Gold (III) porphyrins [35]
LFQP	Straightforward and cost-effective.	It requires more rigorous analytical measurements and statistical validation than isotope-coded measurements.	Cisplatin [47] [Pd(sac)(terpy)](sac) [48] Plecstatin [49] RAPTA agent [50]
2-DE MS	It resolves thousands of intact protein species in a single run.	Time-consuming and labor-intense.	Cisplatin [51] Auranofin [52] Auoxo6 [52] Gold (III) NHC complexes [53]

## Abbreviations

ESI	electron spray ionization
MALDI	matrix assisted laser desorption ionization
TOF	time of flight
HCD	higher energy collisional dissociation
ICAT	isotope-coded affinity tag
ITRAQ	isobaric tag for relative and absolute quantitation
TMT	tandem mass tags
SILAC	stable isotope labeling by amino acids in cell culture
LFQP	label-free quantitative proteomics
2-DE	two-dimensional (2D) gel electrophoresis
HMGB1	high mobility group box 1 protein
PC4	positive cofactor 4
DDB-1	DNA damage-binding protein 1
DJ-1	Protein/nucleic acid deglycase DJ-1
XRCC5	X-ray repair cross-complementing protein 5
2-D DIGE	Two-dimensional fluorescence difference gel electrophoresis
ALDH	aldehyde dehydrogenase
ETF	electron transfer flavoprotein
PHB	prohibitin
PRDX3	peroxiredoxin III
EGFR	epidermal growth factor receptor

## References

[1] Rosenberg B., VanCamp L., Trosko J.E., Mansour V.H. (1969). Platinum compounds: A new class of potent antitumour agents. Nature.

[2] Wang X., Guo Z. (2007). The Role of Sulfur in Platinum Anticancer Chemotherapy. Anticancer Agents Med. Chem..

[3] Meijer C., Mulder N.H., Timmer-Bosscha H., Sluiter W.J., Meersma G.J., de Vries E.G. (1992). Relationship of cellular glutathione to the cytotoxicity and resistance of seven platinum compounds. Cancer Res..

[4] Hall M.D., Okabe M., Shen D.-W., Liang X.-J., Gottesman M.M. (2008). The role of cellular accumulation in determining sensitivity to platinum-based chemotherapy. Annu. Rev. Pharmacol. Toxicol..

[5] Zwelling L.A., Anderson T., Kohn K.W. (1979). DNA-protein and DNA interstrand cross-linking by cis- and trans-platinum(II) diamminedichloride in L1210 mouse leukemia cells and relation to cytotoxicity. Cancer Res..

[6] Jamieson E.R., Lippard S.J. (1999). Structure, recognition, and processing of cisplatin-DNA adducts. Chem. Rev..

[7] Jung Y., Lippard S.J. (2007). Direct cellular responses to platinum-induced DNA damage. Chem. Rev..

[8] Desoize B., Madoulet C. (2002). Particular aspects of platinum compounds used at present in cancer treatment. Crit. Rev. Oncol. Hemat..

[9] Kartalou M., Essigmann J.M. (2001). Recognition of cisplatin adducts by cellular proteins. Mutat. Res. Fundam. Mol. Mech. Mutag..

[10] Farrell N.P. (2015). Multi-platinum anti-cancer agents. Substitution-inert compounds for tumor selectivity and new targets. Chem. Soc. Rev..

[11] Mangrum J.B., Farrell N.P. (2010). Excursions in polynuclear platinum DNA binding. Chem. Commun..

[12] Farrell N.P. (2012). Progress in platinum-derived drug development. Drugs Future.

[13] Manzotti C., Pratesi G., Menta E., Di Domenico R., Cavalletti E., Fiebig H.H., Kelland L.R., Farrell N., Polizzi D., Supino R. (2000). BBR 3464: A novel triplatinum complex, exhibiting a preclinical profile of antitumor efficacy different from cisplatin. Clin. Cancer Res..

[14] Oehlsen M.E., Qu Y., Farrell N. (2003). Reaction of polynuclear platinum antitumor compounds with reduced glutathione studied by multinuclear (H-1, H-1-N-15 gradient heteronuclear single-quantum coherence, and Pt-195) NMR spectroscopy. Inorg. Chem..

[15] Vacchina V., Torti L., Allievi C., Lobinski R. (2003). Sensitive species-specific monitoring of a new triplatinum anti-cancer drug and its potential related compounds in spiked human plasma by cation-exchange HPLC-ICP-MS. J. Anal. At. Spectrom..

[16] Fang T., Cao K., Cheng L., Zhao L., Liu Y. (2017). Protein interaction in the mechanism of platinum anticancer drugs. Sci. Sin. Chim..

[17] Cini M., Bradshaw T.D., Woodward S. (2017). Using titanium complexes to defeat cancer: The view from the shoulders of titans. Chem. Soc. Rev..

[18] Hanif M., Babak M.V., Hartinger C.G. (2014). Development of anticancer agents: Wizardry with osmium. Drug Discov. Today.

[19] Hanif M., Hartinger C.G. (2018). Anticancer metallodrugs: Where is the next cisplatin?. Future Med. Chem..

[20] Liu Z., Sadler P.J. (2014). Organoiridium Complexes: Anticancer Agents and Catalysts. Acc. Chem. Res..

[21] Bergamo A., Sava G. (2011). Ruthenium anticancer compounds: Myths and realities of the emerging metal-based drugs. Dalton Trans..

[22] Pal M., Nandi U., Mukherjee D. (2018). Detailed account on activation mechanisms of ruthenium coordination complexes and their role as antineoplastic agents. Eur. J. Med. Chem..

[23] Bergamo A., Masi A., Dyson P.J., Sava G. (2008). Modulation of the metastatic progression of breast cancer with an organometallic ruthenium compound. Int. J. Oncol..

[24] Scolaro C., Bergamo A., Brescacin L., Delfino R., Cocchietto M., Laurenczy G., Geldbach T.J., Sava G., Dyson P.J. (2005). In vitro and in vivo evaluation of ruthenium(II)-arene PTA complexes. J. Med. Chem..

[25] Wu B., Ong M.S., Groessl M., Adhireksan Z., Hartinger C.G., Dyson P.J., Davey C.A. (2011). A Ruthenium Antimetastasis Agent Forms Specific Histone Protein Adducts in the Nucleosome Core. Chem. Eur. J..

[26] Adhireksan Z., Davey G.E., Campomanes P., Groessl M., Clavel C.M., Yu H., Nazarov A.A., Yeo C.H.F., Ang W.H., Droege P. (2014). Ligand substitutions between ruthenium-cymene compounds can control protein versus DNA targeting and anticancer activity. Nat. Commun..

[27] Jing Y.K., Dai J., Chalmers-Redman R.M.E., Tatton W.G., Waxman S. (1999). Arsenic trioxide selectively induces acute promyelocytic leukemia cell apoptosis via a hydrogen peroxide-dependent pathway. Blood.

[28] Zheng Y.H., Yamaguchi H., Tian C.J., Lee M.W., Tang H., Wang H.G., Chen Q. (2005). Arsenic trioxide (As_2_O_3_) induces apoptosis through activation of Bax in hematopoietic cells. Oncogene.

[29] Englinger B., Pirker C., Heffeter P., Terenzi A., Kowol C.R., Keppler B.K., Berger W. (2018). Metal Drugs and the Anticancer Immune Response. Chem. Rev..

[30] Liu J.X., Zhou G.B., Chen S.J., Chen Z. (2012). Arsenic compounds: Revived ancient remedies in the fight against human malignancies. Curr. Opin. Chem. Biol..

[31] Wang Y., Wang H., Li H., Sun H. (2015). Metallomic and metalloproteomic strategies in elucidating the molecular mechanisms of metallodrugs. Dalton Trans..

[32] Chau D., Ng K., Chan T.S.-Y., Cheng Y.-Y., Fong B., Tam S., Kwong Y.-L., Tse E. (2015). Azacytidine sensitizes acute myeloid leukemia cells to arsenic trioxide by up-regulating the arsenic transporter aquaglyceroporin 9. J. Hematol. Oncol..

[33] Torka P., Al Ustwani O., Wetzler M., Wang E.S., Griffiths E.A. (2016). Swallowing a bitter pill-oral arsenic trioxide for acute promyelocytic leukemia. Blood Rev..

[34] Eisler R. (2003). Chrysotherapy: A synoptic review. Inflamm. Res..

[35] Hu D., Liu Y., Lai Y.-T., Tong K.-C., Fung Y.-M., Lok C.-N., Che C.-M. (2016). Anticancer Gold(III) Porphyrins Target Mitochondrial Chaperone Hsp60. Angew. Chem. Int. Ed..

[36] Saggioro D., Rigobello M.P., Paloschi L., Folda A., Moggach S.A., Parsons S., Ronconi L., Fregona D., Bindoli A. (2007). Gold(III)—Dithiocarbamato complexes induce cancer cell death triggered by thioredoxin redox system inhibition and activation of ERK pathway. Chem. Biol..

[37] Nobili S., Mini E., Landini I., Gabbiani C., Casini A., Messori L. (2010). Gold Compounds as Anticancer Agents: Chemistry, Cellular Pharmacology, and Preclinical Studies. Med. Res. Rev..

[38] Mcluckey S.A. (1992). Principles of collisional activation in analytical mass spectrometry. J. Am. Soc. Mass. Spectrom..

[39] Olsen J.V., Macek B., Lange O., Makarov A., Horning S., Mann M. (2007). Higher-energy C-trap dissociation for peptide modification analysis. Nat. Methods.

[40] Jedrychowski M.P., Huttlin E.L., Haas W., Sowa M.E., Rad R., Gygi S.P. (2011). Evaluation of HCD- and CID-type Fragmentation Within Their Respective Detection Platforms for Murine Phosphoproteomics. Mol. Cell. Proteomics.

[41] Zubarev R.A., Zubarev A.R., Savitski M.M. (2008). Electron capture/transfer versus collisionally activated/induced dissociations: Solo or duet?. J. Am. Soc. Mass. Spectrom..

[42] Toyo’oka T. (2012). LC-MS determination of bioactive molecules based upon stable isotope-coded derivatization method. J. Pharm. Biomed. Anal..

[43] Stewart J.J., White J.T., Yan X.W., Collins S., Drescher C.W., Urban N.D., Hood L., Lin B.Y. (2006). Proteins associated with cisplatin resistance in ovarian cancer cells identified by quantitative proteomic technology and integrated with mRNA expression levels (vol 5, pg 433, 2006). Mol. Cell. Proteomics.

[44] Wang Y., Zhang L., Zheng X., Zhong W., Tian X., Yin B., Tian K., Zhang W. (2016). Long non-coding RNA LINC00161 sensitises osteosarcoma cells to cisplatin-induced apoptosis by regulating the miR-645-IFIT2 axis. Cancer Lett..

[45] Jung J.H., You S., Oh J.W., Yoon J., Yeon A., Shahid M., Cho E., Sairam V., Park T.D., Kim K.P. (2018). Integrated proteomic and phosphoproteomic analyses of cisplatin-sensitive and resistant bladder cancer cells reveal CDK2 network as a key therapeutic target. Cancer Lett..

[46] Chavez J.D., Hoopmann M.R., Weisbrod C.R., Takara K., Bruce J.E. (2011). Quantitative Proteomic and Interaction Network Analysis of Cisplatin Resistance in HeLa Cells. PLoS ONE.

[47] Piskareva O., Harvey H., Nolan J., Conlon R., Alcock L., Buckley P., Dowling P., O’Sullivan F., Bray I., Stallings R.L. (2015). The development of cisplatin resistance in neuroblastoma is accompanied by epithelial to mesenchymal transition in vitro. Cancer Lett..

[48] Adiguzel Z., Baykal A.T., Kacar O., Yilmaz V.T., Ulukaya E., Acilan C. (2014). Biochemical and Proteomic Analysis of a Potential Anticancer Agent: Palladium(II) Saccharinate Complex of Terpyridine Acting through Double Strand Break Formation. J. Proteome Res..

[49] Meier S.M., Kreutz D., Winter L., Klose M.H.M., Cseh K., Weiss T., Bileck A., Alte B., Mader J.C., Jana S. (2017). An Organoruthenium Anticancer Agent Shows Unexpected Target Selectivity for Plectin. Angew. Chem. Int. Ed..

[50] Babak M.V., Meier S.M., Huber K.V.M., Reynisson J., Legin A.A., Jakupec M.A., Roller A., Stukalov A., Gridling M., Bennett K.L. (2015). Target profiling of an antimetastatic RAPTA agent by chemical proteomics: Relevance to the mode of action. Chem. Sci..

[51] Dai Z., Yin J., He H., Li W., Hou C., Qian X., Mao N., Pan L. (2010). Mitochondrial comparative proteomics of human ovarian cancer cells and their platinum-resistant sublines. Proteomics.

[52] Guidi F., Landini I., Puglia M., Magherini F., Gabbiani C., Cinellu M.A., Nobili S., Fiaschi T., Bini L., Mini E. (2012). Proteomic analysis of ovarian cancer cell responses to cytotoxic gold compounds. Metallomics.

[53] Fung S.K., Zou T., Cao B., Lee P.-Y., Fung Y.M.E., Hu D., Lok C.-N., Che C.-M. (2017). Cyclometalated Gold(III) Complexes Containing N-Heterocyclic Carbene Ligands Engage Multiple Anti-Cancer Molecular Targets. Angew. Chem. Int. Ed..

[54] Han D.K., Eng J., Zhou H.L., Aebersold R. (2001). Quantitative profiling of differentiation-induced microsomal proteins using isotope-coded affinity tags and mass spectrometry. Nat. Biotechnol..

[55] Li C., Hong Y., Tan Y.X., Zhou H., Ai J.H., Li S.J., Zhang L., Xia Q.C., Wu J.R., Wang H.Y. (2004). Accurate qualitative and quantitative proteomic analysis of clinical hepatocellular carcinoma using laser capture microdissection coupled with isotope-coded affinity tag and two-dimensional liquid chromatography mass spectrometry. Mol. Cell. Proteomics.

[56] Chen R., Sheng P., Yi E.C., Donohoe S., Bronner M.P., Potter J.D., Goodlett D.R., Aebersold R., Brentnall T.A. (2006). Quantitative proteomic profiling of pancreatic cancer juice. Proteomics.

[57] Gygi S.P., Rist B., Gerber S.A., Turecek F., Gelb M.H., Aebersold R. (1999). Quantitative analysis of complex protein mixtures using isotope-coded affinity tags. Nat. Biotechnol..

[58] Dayon L., Sanchez J.-C., Marcus K. (2012). Relative Protein Quantification by MS/MS Using the Tandem Mass Tag Technology, in Quantitative Methods in Proteomics.

[59] Pierce A., Unwin R.D., Evans C.A., Griffiths S., Carney L., Zhang L., Jaworska E., Lee C.-F., Blinco D., Okoniewski M.J. (2008). Eight-channel iTRAQ enables comparison of the activity of six leukemogenic tyrosine kinases. Mol. Cell. Proteomics.

[60] Casado-Vela J., Jose Martinez-Esteso M., Rodriguez E., Borras E., Elortza F., Bru-Martinez R. (2010). iTRAQ-based quantitative analysis of protein mixtures with large fold change and dynamic range. Proteomics.

[61] Boehm A.M., Pütz S., Altenhöfer D., Sickmann A., Falk M. (2007). Precise protein quantification based on peptide quantification using iTRAQ™. BMC Bioinf..

[62] Ong S.E., Blagoev B., Kratchmarova I., Kristensen D.B., Steen H., Pandey A., Mann M. (2002). Stable isotope labeling by amino acids in cell culture, SILAC, as a simple and accurate approach to expression proteomics. Mol. Cell. Proteomics.

[63] Bicho C.C., Alves F.D., Chen Z.A., Rappsilber J., Sawin K.E. (2010). A Genetic Engineering Solution to the “Arginine Conversion Problem” in Stable Isotope Labeling by Amino Acids in Cell Culture (SILAC). Mol. Cell. Proteomics.

[64] Ong S.-E., Mann M. (2007). A practical recipe for stable isotope labeling by amino acids in cell culture (SILAC). Nat. Protoc..

[65] Kito K., Ito T. (2008). Mass spectrometry-based approaches toward absolute quantitative proteomics. Curr. Genomics.

[66] Kalra H., Adda C.G., Liem M., Ang C.-S., Mechler A., Simpson R.J., Hulett M.D., Mathivanan S. (2013). Comparative proteomics evaluation of plasma exosome isolation techniques and assessment of the stability of exosomes in normal human blood plasma. Proteomics.

[67] Abdallah C., Dumas-Gaudot E., Renaut J., Sergeant K. (2012). Gel-based and gel-free quantitative proteomics approaches at a glance. Int. J. Plant Genomics.

[68] Anand S., Samuel M., Ang C.-S., Keerthikumar S., Mathivanan S. (2017). Label-Based and Label-Free Strategies for Protein Quantitation. Methods Mol. Biol..

[69] Zhang Y., Wen Z., Washburn M.P., Florens L. (2015). Improving Label-Free Quantitative Proteomics Strategies by Distributing Shared Peptides and Stabilizing Variance. Anal. Chem..

[70] Chiara G., Francesca M., Alessandra M., Luigi M. (2010). Proteomic and Metallomic Strategies for Understanding the Mode of Action of Anticancer Metallodrugs. Anticancer Agents Med. Chem..

[71] Cho Y.-E., Singh T.S.K., Lee H.-C., Moon P.-G., Lee J.-E., Lee M.-H., Choi E.-C., Chen Y.-J., Kim S.-H., Baek M.-C. (2012). In-depth Identification of Pathways Related to Cisplatin-induced Hepatotoxicity through an Integrative Method Based on an Informatics-assisted Label-free Protein Quantitation and Microarray Gene Expression Approach. Mol. Cell. Proteomics.

[72] Du Z., Luo Q., Yang L., Bing T., Li X., Guo W., Wu K., Zhao Y., Xiong S., Shangguan D. (2014). Mass Spectrometric Proteomics Reveals that Nuclear Protein Positive Cofactor PC4 Selectively Binds to Cross-Linked DNA by a trans-Platinum Anticancer Complex. J. Am. Chem. Soc..

[73] Tušek-Božić L., Furlani A., Scarcia V., De Clercq E., Balzarini J. (1998). Spectroscopic and biological properties of palladium(II) complexes of ethyl 2-quinolylmethylphosphonate. J. Inorg. Biochem..

[74] Kim W.T., Kim J., Yan C., Jeong P., Choi S.Y., Lee O.J., Chae Y.B., Yun S.J., Lee S.C., Kim W.J. (2014). S100A9 and EGFR gene signatures predict disease progression in muscle invasive bladder cancer patients after chemotherapy. Ann. Oncol..

[75] Chatterjee S., Kundu S., Bhattacharyya A., Hartinger C.G., Dyson P.J. (2008). The ruthenium(II)-arene compound RAPTA-C induces apoptosis in EAC cells through mitochondrial and p53-JNK pathways. J. Biol. Inorg. Chem..

